# Swyer-James-MacLeod Syndrome in an East African Patient

**DOI:** 10.7759/cureus.69874

**Published:** 2024-09-21

**Authors:** Rachel E Berndsen, Mark E Montgomery, Kaya Belknap, Cristóbal S Berry-Cabán

**Affiliations:** 1 Family Medicine, Campbell University School of Osteopathic Medicine, Lillington, USA; 2 Family Medicine, AIC Kijabe Hospital, Kijabe, KEN; 3 Epidemiology, Womack Army Medical Center, Fayetteville, USA

**Keywords:** bronchiectasis, bronchiolitis, hyperlucent lung syndrome, pulmonary emphysema, swyer-james-macleod syndrome, unilateral pulmonary hypoplasia

## Abstract

Swyer-James-MacLeod syndrome (SJMS) is a rare pulmonary disorder characterized by unilateral hypoplasia of pulmonary vasculature, often resulting in emphysema and sometimes bronchiectasis. Although its exact cause remains uncertain, SJMS is believed to be a complication of childhood respiratory infections, such as those caused by respiratory syncytial virus, influenza, Bordetella pertussis, and Mycobacterium tuberculosis. The condition often presents with nonspecific respiratory symptoms, making diagnosis challenging and frequently delayed. We report a case of a 23-year-old Kenyan female with a complex clinical history, including prior tuberculosis treatment and multiple clinic visits over 17 months for symptoms of dyspnea, cough, and weight loss. These nonspecific symptoms made narrowing the differential diagnosis difficult and resulted in a long clinical course. Conservative management remains the primary approach for most SJMS patients; however, surgical intervention may be necessary in cases of recurrent infections or severe pulmonary complications. This case underscores the importance of considering SJMS in patients with unexplained respiratory symptoms and highlights the need for thorough evaluation and advanced imaging.

## Introduction

Swyer-James-MacLeod syndrome (SJMS), also known as Swyer-James syndrome or unilateral hyperlucent lung syndrome, is characterized by abnormal development of the affected lung or part of the lung, resulting in it being slightly smaller than the opposite lung. SJMS is a rare lung disorder distinguished by unilateral functional hypoplasia of pulmonary vasculature and emphysema, with or without associated bronchiectasis [1].

The exact cause of this syndrome remains unclear, but it is believed to be a rare pulmonary complication of childhood respiratory infections, including viruses such as respiratory syncytial virus (RSV), influenza A and bacteria such as Bordetella pertussis and Mycobacterium tuberculosis [1].

One proposed mechanism for the development of SJMS involves bronchiolitis caused by the mentioned respiratory infections with obliteration of peripheral bronchial branches [2]. The exact incidence of SJMS is not well-established, as it is considered a rare condition. However, it is estimated to occur in fewer than one in 100,000 individuals [3]. Because it often goes undiagnosed or misdiagnosed, due to the highly variable symptoms, the true prevalence may be higher than reported. It is more commonly diagnosed in childhood but can also be identified in adults. One study reported SJMS to be present in 0.01% in a survey of 17,450 chest radiographs [2]. Patients may display symptoms of cough and/or hemoptysis or dyspnea on exertion, though many experience no symptoms [2]. The variable presentation can cause delayed diagnosis, or it may even be discovered as an incidental finding on chest X-rays.

## Case presentation

A 23 year-old Kenyan female presented to the clinic multiple times over the span of 17 months with initial symptoms of dyspnea at rest, cough, and weight loss. Past medical history includes treatment for primary tuberculosis one year prior with three thoracocenteses, convulsions, and a benign left breast mass.

**Figure 1:**
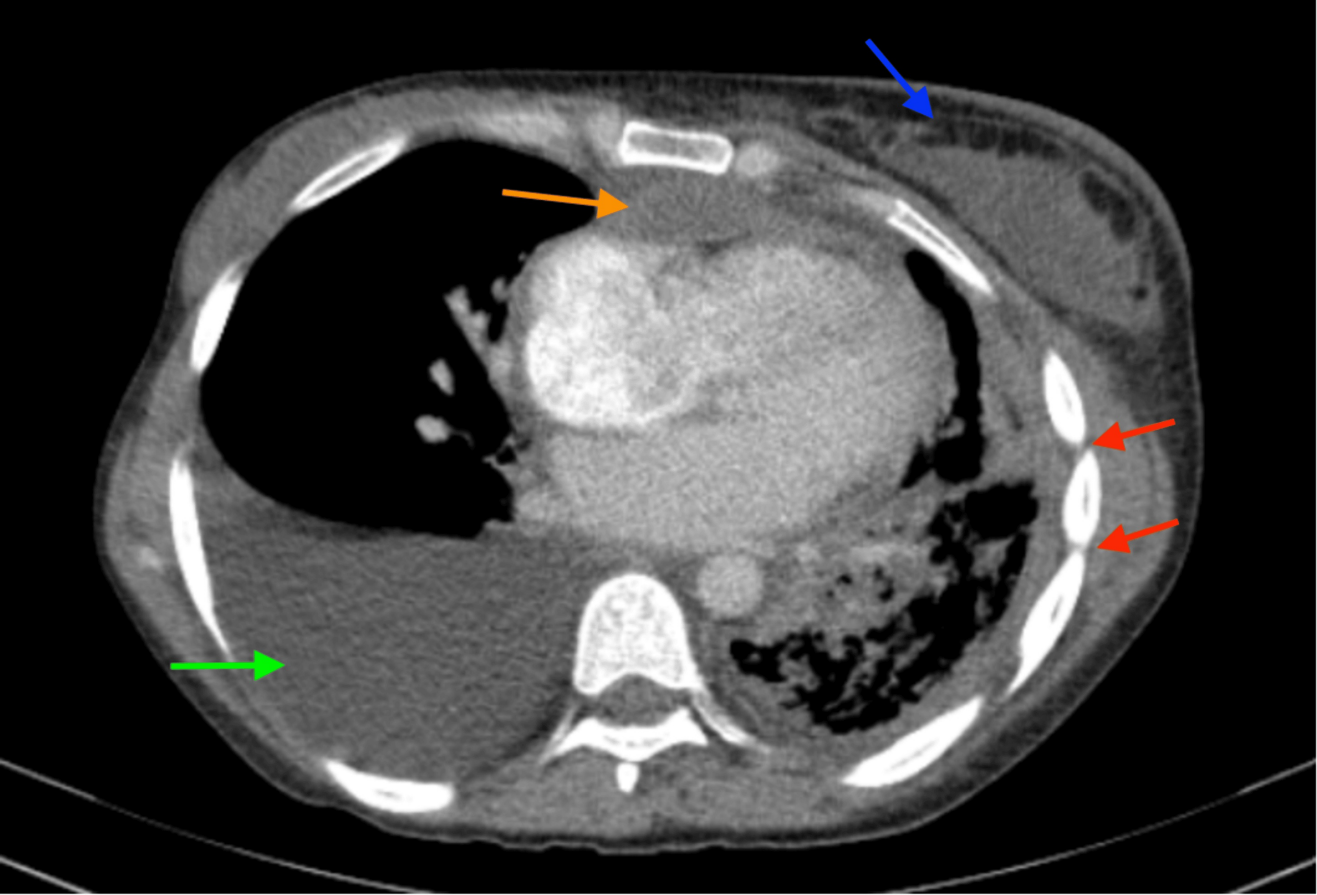
Chest CT showing decreased spacing between ribs on the left (red arrows), a large right-sided pleural effusion (green arrow), pericardial mass (orange arrow), and left breast mass (blue arrow).

Physical exam revealed reduced breath sounds on the left, crackles in the right lower lung, and an enlarged left breast. Initial chest computed tomography (CT) scan showed a large right pleural effusion, decreased left lung size with reticulonodular opacities, features of a left lung abscess, and a pericardial effusion (Figure 1). Magnetic resonance imaging (MRI) of the head showed bilateral ring enhancing lesions at the gray-white junctions concerning for neurocysticercosis.

The patient was treated for neurocysticercosis with albendazole 15 mg/kg/day for 14 days and dexamethasone 0.1 mg/kg/day. This diagnosis was made based on brain MRI showing multiple ring-enhancing lesions and bilateral cerebral hemispheres. and bacterial pneumonia and had a thoracentesis that drained 1L of fluid that was suggestive of a reactive pleural effusion. She was lost to follow up for 10 months.

The patient returned to the clinic due to worsening edema and notable weight loss. Chest-abdomen CT showed bilateral pleural effusions, pericardial mass, congested and enlarged liver measuring 15.1 cm. An echocardiogram revealed dilation of the right atrium and inferior vena cava (IVC), thickened pericardium, and decreased right ventricular lumen size suggestive of constrictive pericarditis.

**Figure 2:**
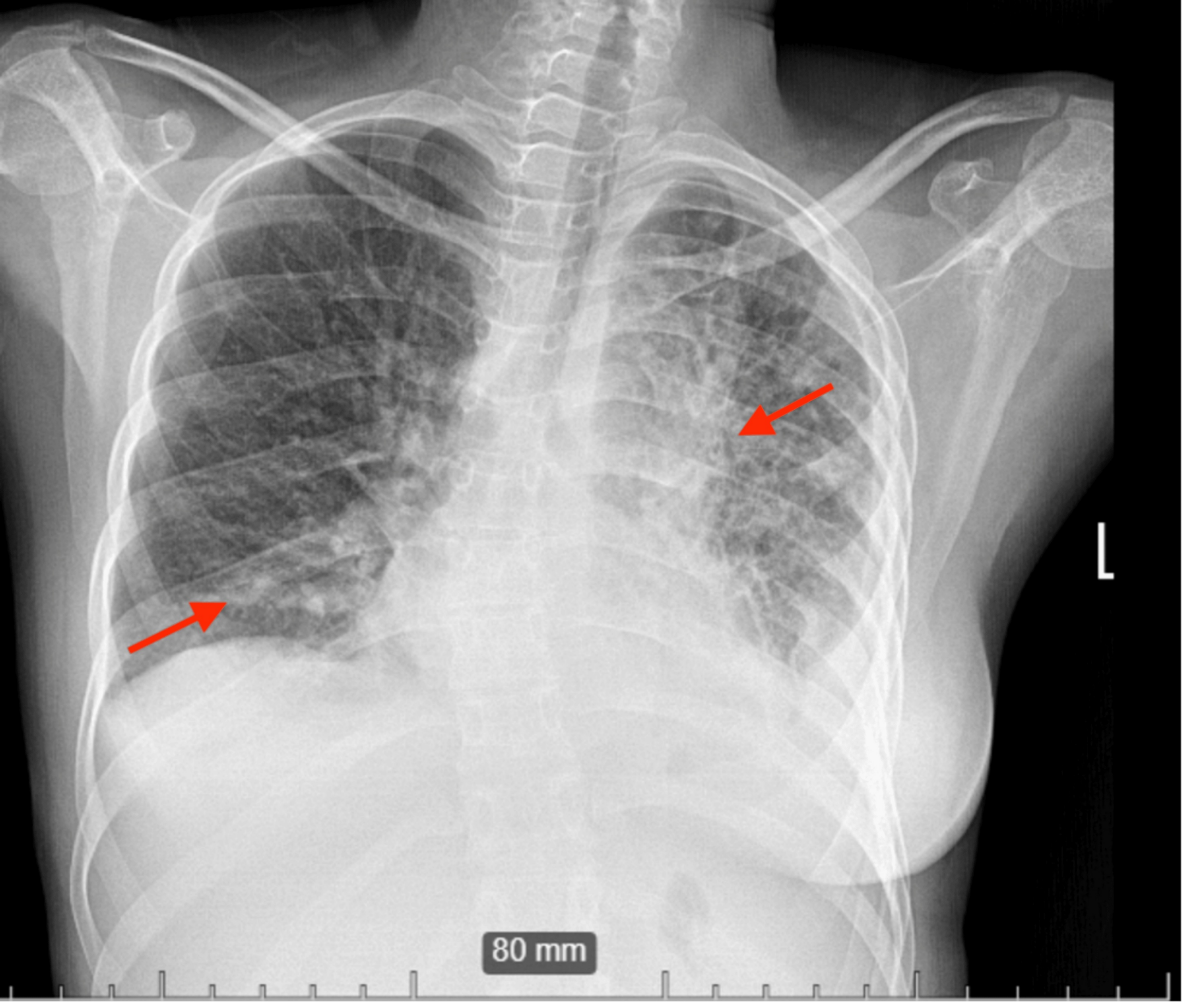
Chest X-ray showing left lung hypoplasia and bilateral interstitial infiltrates (red arrows).

**Figure 3:**
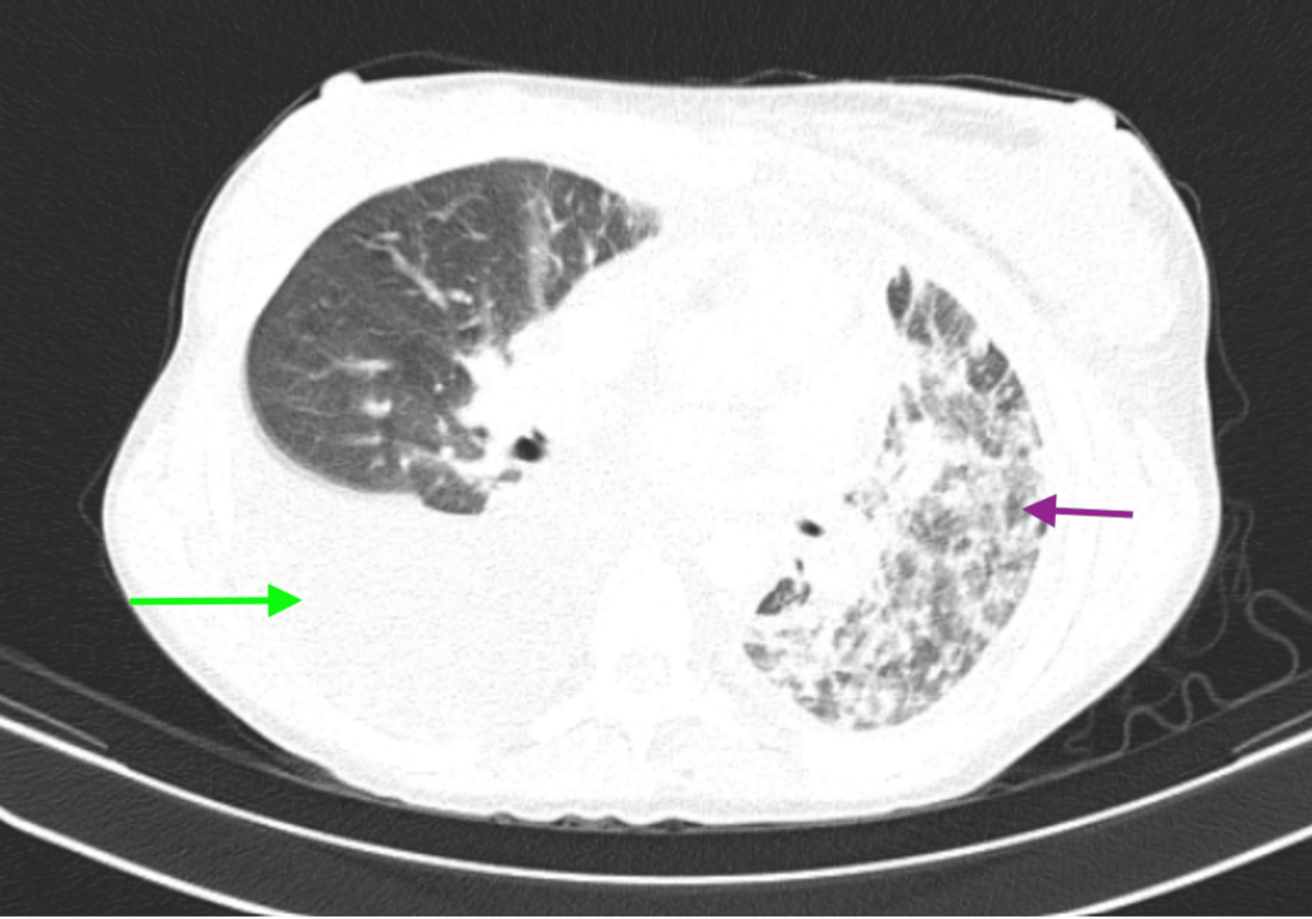
CT scan showing large right-sided pleural effusion (green arrow) and abnormal left-sided parenchyma (purple arrow).

She was eventually diagnosed with SJMS as a result of imaging findings (Figures 2, 3) and clinical course.

Since being diagnosed the exact cause of her symptoms is still unknown. A sputum sample was collected and sent for GeneXpert (Cepheid, Sunnyvale, CA, USA) testing and the patient was empirically started on a second course of antitubercular medication including rifampicin, isoniazid, pyrazinamide, ethambutol (RHZE), pyridoxine, and prednisolone for suspected extrapulmonary tuberculosis but four months into the treatment her symptoms continued to worsen. Local experts postulated that her difficulty breathing might be due to the pericardial mass, she is currently awaiting thoracotomy and removal of the pericardial mass to aid in diagnosis and to improve her heart failure symptoms.

## Discussion

Diagnosing SJMS is challenging due to several factors including the rarity of the condition [1]; the symptoms of SJMS, such as chronic cough, recurrent respiratory infections, and shortness of breath, are non-specific and can overlap with many other more common respiratory conditions like asthma, chronic obstructive pulmonary disease (COPD), or bronchiectasis [4]; in the early stages, the radiological findings of SJMS can be overlooked; the variability in presentation and lastly the overlap with other respiratory conditions, such as bronchiectasis or infections that can obscure the primary diagnosis [1,5].

Diagnosis in this patient took over a year of clinic visits and many diagnostic tests. SJMS is considered an acquired syndrome caused by infectious insult to the lungs [6]. This is consistent with the patient’s history of tuberculosis, which is fairly common in East Africa [7-9]. Delays in diagnosis can cause erroneous treatment for other conditions such as asthma or COPD with corticosteroids, which may leave SJMS patients more susceptible to pulmonary infections [6]. With this in mind, a variety of tests and images can aid in the diagnosis of SJMS when working up a patient with a history of recalcitrant pulmonary symptoms and infections. Clinical history, ventilation/perfusion (V/Q) scans, high-resolution CT scans, and pulmonary function tests have been proposed as tools to narrow a diagnosis to SJMS [10]. Pulmonary function tests usually demonstrate a restrictive pattern and a significant reduction in vital capacity [10]. Additionally, a delay in diagnosing chronic conditions like this can exacerbate psychological distress regarding one's health, a phenomenon observed in our patient.

Treatment of SJMS involves close follow-up on patients to manage recurrent pulmonary infections, though some cases may be managed surgically if infections and/or pneumothorax cannot be controlled [11]. Pneumonectomy has been shown to be an effective treatment for improving pulmonary function and clinical symptoms of SJMS in treatment-refractory cases [11].

Patients with SJMS may either remain asymptomatic over long periods or experience recurrent pulmonary infections [1]. Typically, conservative management is the primary approach for the majority of SJMS patients. However, spontaneous pneumothorax is regarded as a critical situation, especially in individuals with SJMS, given the underlying pathological condition. Surgical intervention is warranted when there is a heightened recurrence of lung infections, compression atelectasis due to emphysematous lung affecting healthy lung tissue, or dyspnea from shunting. The recommended treatment in such cases involves pneumonectomy or lobectomy if hyperinflation affects an entire lung or lobe.

## Conclusions

This case of SJMS highlights the complexities in diagnosing and managing a rare pulmonary disorder. The patient's protracted clinical course, marked by delayed identification of SJMS, underscores the importance of thorough evaluation in patients with persistent respiratory symptoms. Advanced imaging techniques and a detailed clinical history are crucial in distinguishing SJMS from more common respiratory conditions. Despite the challenges in management, including ongoing symptoms and the need for potential surgical intervention, this case emphasizes the need for heightened awareness and early diagnosis to improve outcomes. Clinicians should consider SJMS in differential diagnoses, especially in patients with a history of respiratory infections, to prevent unnecessary treatments and to address the condition more effectively.
